# Peer Victimization and Onset of Social Anxiety Disorder in Children and Adolescents

**DOI:** 10.3390/brainsci9060132

**Published:** 2019-06-06

**Authors:** Maria Pontillo, Maria Cristina Tata, Roberto Averna, Francesco Demaria, Prisca Gargiullo, Silvia Guerrera, Maria Laura Pucciarini, Ornella Santonastaso, Stefano Vicari

**Affiliations:** Child and Adolescence Neuropsychiatry Unit, Department of Neuroscience, Children Hospital Bambino Gesù, Piazza Sant’Onofrio 4, 00165 Rome, Italy; mariacristina.tata@opbg.net (M.C.T.); roberto.averna@opbg.net (R.A.); francesco.demaria@opbg.net (F.D.); prisca.gargiullo@opbg.net (P.G.); silvia.guerrera@opbg.net (S.G.); laura.pucciarini@gmail.com (M.L.P.); ornella.santonastaso@libero.it (O.S.); stefano.vicari@opbg.net (S.V.)

**Keywords:** peer victimization, social anxiety, bullying, adolescents, children

## Abstract

Background: In the literature, several studies have proposed that children and adolescents with social anxiety had experienced previously victimization from peers and siblings. The aim of this review was to contribute to the updating of recent findings about the relationship between peer victimization and onset of social anxiety in children and adolescents. Methods: A selective review of literature published between 2011 and 2018 on Social Anxiety Disorder in children and adolescents that experienced peer victimization during childhood and adolescence. Results: Seventeen studies are included. All studies showed that peer victimization is positively correlated to the presence of social anxiety. Moreover, the perpetration of peer victimization may contribute to the maintenance and the exacerbation of social anxiety symptoms. Conclusions: In children and adolescents with Social Anxiety Disorder, it is necessary to evaluate firstly the presence of peer victimization experiences. Subsequently, therapeutics programs targeted to elaborate these experiences and to reduce the anticipatory anxiety and the avoidance that characterized these children and adolescents can be proposed.

## 1. Introduction

Peer victimization is a physical, verbal or relational aggressive experience of negative or aggressive act by peers [1,2]. It is intentional and repeated versus a victim who cannot protect him or herself [3]. Peer victimization can be divided into direct, overt or active victimization, when the act is direct and aggressive and the victim is physically mistreated, warned or verbally attacked; relational or passive victimization, when the behaviors are targeted at damaging friendships and peer relations. They include exclusion, withdrawing friendship, and gossiping. 

The most severe manifestation of peer victimization is bullying, characterized by an asymmetric power relationship between bully and victim, which may be physical or psychological [4]. Another recent form of victimization is cyberbullying, which the aggressions is entirely online and using electronic media [5].

During peer victimization, involved children and adolescents assume different roles: *bullies*, children or adolescents that victimized other peers; *victims*, those who were victimized; *bully-victims*, children and adolescents who are contemporary bully and victims. These roles are involved directly in the victimization. There are also indirect involvement people, defined *bystanders*, that are children and adolescent that assist to the victimization. For example, the *assistant of the bully* or the *outsider*, that reinforce the negative behavior of the bully, and the *victim defender* that could help the victims [2,6,7,8].

Inchley et al. [9], in collaboration with The Health Behaviour in School-Aged Children (HBSC), conducted an international cross-sectional survey to investigated young’s people health from a nationally representative sample of schools (220.000 students, 11 years old-examined also at 13 and 15 years) in 42 countries in Europe and North America in 2013–2014. The results showed that the overall prevalence of peer victimization was approximately 12% for boys and 10% for girls. 

Furthermore, the prevalence of peer victimization decreased with age, peaking for boys at 11 and reach the lowest level at 15. Instead, levels for girls were more constant at ages 11 and 13 and declined at 15 [9].

During adolescence, the approval of peers is increasingly emphasized and peer victimization could have a greater psychological impact on young people [10,11,12]. Indeed, in childhood and adolescence, peer victimization are frequently associated with internalizing problems, as anxiety, depression and a decreasing of self-worth and self-esteem [13,14]. In particular, repeated peer victimization could cause significant distress, hypervigilance, fear of future attacks and self-blame [15,16]. These symptoms could increase social avoidance, isolation and loneliness that could degenerate in a frank disorder [17]. For example, the anxiety for social relationship and the consequent avoidance constitute the core symptoms of the Social Anxiety Disorder (SAD), or Social Phobia (according to the DSM-IV-TR) [18]. According to the DSM-5 (Diagnostic and Statistical Manual of Mental Disorders) [19], SAD is a disorder characterized by a significant anxiety or fear about social situations in which the children or the adolescent could be exposed to comparison with peers or adults. This fear provoke an avoidance of social situation and the reaction is out of proportion to the actual danger, that could be expressed by crying, tantrums, freezing, clinging, shrinking, or failing to speak. To diagnose a Social Anxiety Disorder this symptoms must be persistent, typically lasting for 6 months or more, and cause significant impairment in social, familiar or scholar functioning [19]. Lifetime prevalence rates of SAD in children and adolescents range from 1.6% to 13.3% [20,21,22,23].

Several studies had investigated the relationship between peer victimization and social anxiety. Findings showed the presence of various psychiatric symptoms in children and adolescents with peer victimization experiences [24,25,26,27,28,29,30,31,32,33]. In fact, victims of different forms of peer victimization (verbal, physical and relational), presented higher level of social anxiety [34,35,36,37]. In a longitudinal perspective, the relationship between social anxiety and peer victimization could be explained in a negative circle: socially anxious children may be at risk for peer victimization and perpetration of peer victimization may generate higher levels of social anxiety [34].

The primary aim of our study was to investigate in the recent literature the possible link between peer victimization and the onset of social anxiety. The secondary aim of our review was to analyze possible psychological factors that could moderate the relationship between peer victimization and social anxiety. Overall, the goal of this review was to focus on the negative consequences of peer victimization on the social and psychological functioning of children and adolescents.

## 2. Materials and Methods

### 2.1. Study Design

This paper consists of a selective review of the literature published between 2011 and 2018. The authors followed the Preferred Reporting Items for Systematic Reviews and Meta-Analyses (PRISMA) guidelines [38].

### 2.2. Search Strategy

A comprehensive literature search of the PubMed/MEDLINE, Cochrane Library, the Cumulative Index to Nursing and Allied Health Literature (CINHAL) and Scopus databases was conducted. A search algorithm based on a combination of these terms was used: (bullying OR peer victimization) AND (social phobia OR Social Anxiety Disorder) AND (children AND adolescents). We used also the keyword “social phobia” because it was the DSM-IV-TR [18] diagnostic label and our search include studies published before the release of DSM-5 [19]. The last update of the search was on January 2019.

### 2.3. Selection Criteria

The included studies investigated the prevalence and the clinical significance of Social Anxiety Disorder and social phobia in children and adolescents that experienced peer victimization during childhood and adolescence. Therefore, we included studies were the focus was primary on peer victimization correlated to internalizing problems, especially social anxiety, experienced during childhood or adolescence. Articles not in the area of interest of this review were excluded (e.g., review articles, conference proceedings, comments, editorials or letters). No language restrictions or study design restrictions were applied.

### 2.4. Selection Procedure, Data Extraction and Data Management

**Figure 1:**
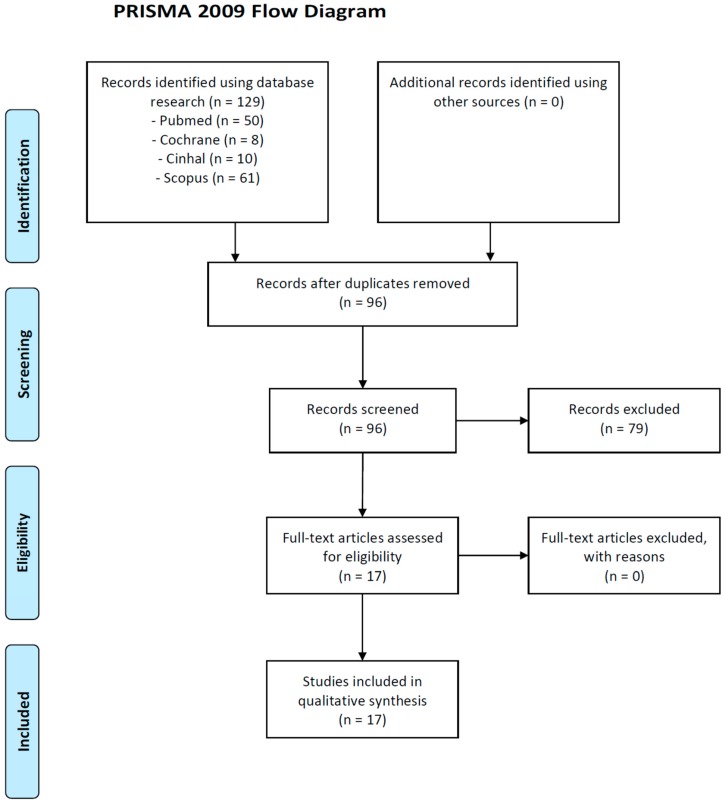
PRISMA 2009 Flow Diagram [37].

The bibliographies of the most relevant published articles in the area of our interest were examined. Data on efficacy, acceptability and tolerability were extracted by six authors independently (R.A., F.D., P.G., S.G., M.L.P. and O.S.). Disagreements were sorted out in a consensus meeting by other reviewers (M.P., S.V. and M.C.T.). The search algorithm resulted in 96 articles. Of these, 79 articles were excluded because not in the field of our research. 17 referred to potentially eligible studies. In terms of evidence-based medicine, the quality of these studies was moderate. Figure 1 presents a detailed flow diagram of the study selection process. Table S1 presented the detailed search strategy process. 

## 3. Data Synthesis

We found a total of seventeen studies on peer victimization and onset of social anxiety disorder in children and adolescents. Due to the number of included studies, we proposed a narrative synthesis. The narrative synthesis required describing, organizing, exploring and interpreting the study findings, examining their methodological adequacy.

## 4. Results

### 4.1. Peer Victimization and Social Anxiety Disorder: Prevalence and Clinical Aspects

During the last seven years, twelve studies have been conducted to investigate the prevalence of peer victimization in children and adolescents with Social Anxiety Disorder. Details on the methodologies and results of the studies are shown in Table 1.

Gren-Landell and colleagues [39] conducted a cross-sectional study in a community sample of 3211 Swedish high-school students (mean age: 17.3). Results showed that 10.6% (*n* = 340) of the sample self-reported symptoms compatible with the presence of SAD and high levels of peer victimization (*p* < 0.001). In particular, females reported significantly more maltreatment, sexual victimization and victimization from peers and siblings (*p* < 0.001) compared to males.

Moreover, Pabian and Vandebosch [40] proposed a short-term longitudinal study in two time-point (time range: 6 months) among 2128 adolescents (mean age: 13.02). Results showed that at baseline social anxiety had a positive correlation with victimization of traditional bullying (*p* < 0.001), victimization of cyberbullying (*p* < 0.001), and perpetration of traditional bullying (*p* < 0.001). Furthermore, these aspects correlated positively with social anxiety 6 months later. Consequently, this study allowed support for the link between victimization and later perpetration, but social anxiety did not moderate this relationship. In fact, adolescents who scored higher on social anxiety had the same possibility to be victimized 6 months later that adolescents who scored lower. 

Cohen and Kendall [41] examined the presence of experience of peer victimization in a sample of 90 adolescents (mean age: 11.06) in treatment for social phobia and other anxiety disorder. Results showed that overt victimization significantly predicted social anxiety (*p* = 0.003) and loneliness (*p* = 0.001) and relational victimization added significantly to the prediction of social anxiety (*p* = 0.002), depressive symptoms (*p* = 0.001), and loneliness (*p* < 0.001).

The aim of Van Oort et al. [47] was to identify the risk factors that exacerbate the levels of anxiety symptoms throughout adolescence at three time-points (across a 5-year interval) in a community sample of 2220 adolescents from The Netherlands. Risk factors analysis included temperament, self-confidence, IQ, proximal family factors (parenting and parental stress), parental psychopathology (past and present), contextual family factors (life event and family dysfunction), global family factors (parents education, number of children, single parent family) and school factors (sociometric status and bullying). Results showed that victimization experience was correlated to the presence of social phobia in late adolescence (*p* < 0.001). Moreover, a moderate correlation with social phobia has been found in correlation with being a bully, experienced parents’ mental and physical health problems and the sociometric status (*p* < 0.05). 

Moreover, to examine the possible differences between direct and relational victimization, Ranta et al. [45] performed a longitudinal study at two time-points (time range: 2 years) in a group of 3278 adolescents (mean age: 15.5). Results evidenced gender differences correlated with the type of victimization. In particular, in boys’ group there is a bidirectional association between direct peer victimization and social phobia (*p* < 0.05). Instead, in girls’ group, results showed that relation victimization predicted the onset of social phobia (*p* < 0.001).

Hamilton and colleagues [43] proposed a longitudinal study at three time-points (time range: 9 months) in a sample of 410 early adolescents (mean age: 12.84). Results showed that peer victimization, associated with interpersonal stressors and emotional maltreatment, predicted social anxiety and depressive symptoms. Moreover, in a longitudinal reading, the presence of peer victimization predicted the onset of depressive symptoms and consequently the presence of social anxiety. Instead, to investigate the possible association between defending behaviour and mental health among different bullying roles, Wu et al. [48] proposed a cross-sectional study in a sample of 2872 Taiwanese students (age range: 13–15). Defending behaviours could be defined as every behaviour acted to stop a bullying action. Results showed that defending behaviours were associated with bullying roles. In fact victims reporting higher defending behaviour scores than bullies or bystanders. Defending behaviours were also positively associated with social anxiety and depressive symptoms in victims and bystanders (*p* = 0.005). Moreover victims and bully-victims also had greater social anxiety (*p* < 0.001) than bullies.

Yen et al. [49] examined the mediating effect of bullying involvement on the relationships between mental health problems and BMI (Body Mass Index) in a sample of 5252 Taiwanese students (age range: 12–18). The results indicated that the severities of victimization of passive, active bullying and perpetration of passive bullying were positively associated with the severities of social phobia, depression and suicidality (*p* < 0.001). In addition, experiencing victimization of bullying may also increase adolescents’ levels of anticipatory anxiety of being bullied again in socially interactional situations, which increases the risk of developing social phobia. In addition, BMI was positively associated with the severities of victimization of passive (*p* < 0.001), active bullying (*p* < 0.01) and perpetration of passive bullying (*p* < 0.05).

In a subsequent study, Yen et al. [50] investigated the association between victimization experiences and the risk or the presence of mental health problems in a sample of 6445 Taiwanese students (age 12–18) randomly selected. The results indicated that being a victim of passive or active bullying and being a perpetrator of passive bullying were significantly associated with mental health problems, including social phobia (*p* < 0.001). In particular, victims of both passive and active bullying had higher risk to develop several dimensions of mental health problems (social phobia: *p* < 0.001; depression: *p* < 0.001; inattention: *p* < 0.001) than victims of only passive or active bullying. Also for the perpetrators of both passive and active bullying the results indicated more severe symptoms of depression and hyperactivity/impulsivity than the ones of only passive or only active type of bullying (*p* < 0.001). Otherwise, perpetrators of only active bullying reported less severe general anxiety and social phobia than perpetrators of only passive bullying (*p* < 0.001).

A particular study on a selected sample of 31 American/Black girls’ sample (mean age: 14) who were from single-parent, low-income households, was conducted by Davis [42] to examine the relationship between the Acting White Accusation, bullying victimization, social anxiety and ethnic/racial identity. The Acting White Accusation arises when a black adolescent’s ethnic/racial identity is perceived as being not Black enough by another Black adolescent or group of adolescents. The results indicated that White accusation (either directly, indirectly, or both), was significantly associated with social anxiety symptoms (*p* < 0.01), especially when it is interpreted as a negative evaluation, which could lead to fears or reluctance to interact with their Black peers. The accusation was also significantly associated with bullying victimization (*p* < 0.05). 

Differently, Rudolph et al. [46] conducted an experimental study, through functional magnetic resonance imaging scan, to investigate the correlation between neural activation and induced social exclusion in a sample of 47 adolescents girls (mean age: 15.46), of which 24 were chronically victimized girls (mean age: 15.46) and 23 were non-victimized girls (mean age: 15.35). Results demonstrated that victimized girls reported higher levels of depressive symptoms (*p* < 0.001), social anxiety (*p* < 0.01), behavioural inhibition (*p* < 0.001) and avoidance-oriented need for approval (*p* < 0.05). Furthermore, they had greater activation of the social pain network, including the dorsal anterior cingulate cortex (dACC), the subgenual anterior cingulate cortex (sgACC) and anterior insula. Each of this three regions was significantly associated with heightened internalizing symptoms (*p* < 0.001). 

Concluding, a recent longitudinal study conducted by Quinlan et al. [44] on 682 participants (mean age: 14.4) showed that chronic peer victimization was associated with steeper decreases in left putamen volume (*p* = 0.037). These changes were also negatively associated with generalized anxiety (*p* = 0.020). This study did not focused specifically on social anxiety, but it is an important beginning to the study of brain modification consequently to environmental factors, such as peer victimization.

### 4.2. Clinical and Psychological Moderators of Relationship between Peer Victimization and Onset of Social Anxiety Disorder in Children and Adolescents

During the last seven years, five studies have been conducted to investigate the clinical and psychological moderators that influenced the relationship between peer victimization and the onset of Social Anxiety Disorder specifically in children and adolescents. Details on the methodologies and results of the studies are shown in Table 2.

Calvete et al. [51] examined the moderating roles of personality factors like neuroticism and extraversion in 1440 adolescents’ victims of bullying (mean age: 13.54). Indeed, according to the stress-diathesis model [52,53,54], Calvete et al. [51] hypothesized that adolescents with high levels of neuroticism and low levels of extraversion would react to victimization with increased symptoms of depression and social anxiety. Psychological constructs of neuroticism and extraversion are defined according to the Big Five Questionnaire-Children [55].

Results showed that neuroticism was correlated with bullying victimization and depression and social anxiety symptoms at T1, T2, T3 (time range: 6 months). According to this, the predictive association between victimization and social anxiety was lower among girls who were high in extraversion than among girls who were low in extraversion, whereas the association was similar in boys who were high and low in extraversion. Moreover, the adolescents with high levels of extraversion presented a greater reduction in depressive symptoms over time than adolescents with low levels. In addition, although neuroticism predicted both depression and social anxiety, no significant interactions were evident between neuroticism and bullying victimization. Regarding gender differences, the association between bullying victimization and social anxiety was stronger for boys than for girls (*p* < 0.001), whereas the association between neuroticism and depression was stronger for girls (*p* < 0.001).

The role of level of self-worth on the relationship between peer victimization and internalizing problems like onset of social anxiety was investigated in Ghoul et al. [57] in a sample of 716 adolescents (mean age: 15.95). Results showed that victimization and contingent self-worth were positively correlated with social phobia (*p* < 0.001). In particular, moderation analyses suggested that higher levels of contingent self-worth amplify the effect of victimization on internalizing problems. For social phobia, this effect appeared to be salient only for boys. 

The aim of the cross-sectional quantitative survey by Early et al. [56] was to investigate the possible association between social anxiety and other anxiety disorder with peer acceptance or victimization in a clinical sample (*n* = 154) compared to a community sample (*n* = 116) of adolescents (mean age: 13.07). Results showed that the social anxiety group presented lowest social acceptance compared to other anxiety group and the community sample. Instead, relational victimization was significantly higher in the community sample than in the other anxiety group (*p* < 0.05). For authors, the SAD group presented lower relational victimization probably because it is less engaged in social interaction and consequently social avoidance may protect adolescents with SAD from these experiences. 

Regarding the genetic and environmental influences on bullying victimization and psychiatric disturbance, Silberg et al. [58] proposed a cohort-sequential, multi-wave longitudinal study with follow-up into young adulthood (aged ≥18 years) in 145 bully-discordant monozygotic (MZ) twin pairs (age range: 8–17 years). 

The results showed that the bullied MZ twins compared to their non-bullied co-twin have the higher rate of social anxiety (27%), separation anxiety (15%), Attention Deficit and Hyperactivity Disorder (5%), and young adult suicidality (12%). They also presented significant individual-specific environmental correlations between bullying victimization and social anxiety, separation anxiety and young adult suicidality. The authors showed a highly significant association between early bullying victimization and later social anxiety (*p* < 0.001) and bullying victimization and later young adult suicidality (*p* < 0.05). Genetic factors were also influential in bullying victimization and social anxiety. 

Concluding, Spence et al. [59] investigated the 12-month prevalence of social anxiety disorder, separation anxiety disorder and generalized anxiety disorder in a representative sample of Australian youth. Participants of this study were 6310 parents and caregivers of eligible households where there was at least one child aged 4–17 years. The results showed that the presence of social anxiety disorder, separation anxiety disorder and generalized anxiety disorder were all associated with having a parent with a mental health problem and repeated bullying over the previous 12 months (*p* < 0.001).

## 5. Discussion

The goal of this selective review was to contribute to the updating of recent findings about the relationship of peer victimization and Social Anxiety Disorder in children and adolescents. In summary, all seventeen studies included showed that the peer victimization is associated to the presence of a symptomatology compatible with Social Anxiety Disorder. This association has been detected both in clinical samples that in community samples. For example, in Gren-Landell et al. [39], socially phobic adolescents showed significantly higher rates of lifetime victimization from peers/siblings than not socially phobic adolescents. This is in line with results of other studies [42,43,44,46,48,49,50,56,58,59,60].

Focusing on gender differences, Gren-Landel et al. [39] and Ranta et al. [45] found interesting results about. In fact, in Gren-Landell et al. [39], females reported significantly more maltreatment, sexual victimization and victimization from peers/siblings compared to males. Furthermore, in Ranta et al. [45], relation victimization in girls predicted the onset of social phobia; instead, in boys was found a bidirectional association between direct peer victimization and social anxiety. These results indicate that boys could have personal characteristics that make them vulnerable to victimization across different contexts, while girls’ victimization may partially be realized in close friendships and so be more context-specific. 

In a longitudinal perspective, Van Oort et al. [47] showed that early victimization experiences were associated to the presence of social anxiety disorder in late adolescence. Pabian and Vandebosch [40] deepen this point, showing that baseline social anxiety is positive correlated with all three dimensions of bullying: traditional bullying, victimization of cyberbullying and perpetration of traditional bullying. This association persisted also six months later, supporting the role of perpetration of bullying as a critical factor for maintenance of social anxiety. 

This result supports that of previous studies for which repeated peer victimization could cause significant distress, hypervigilance, social avoidance, isolation and loneliness [15,16,17] that are typical symptoms of clinical picture associated to social anxiety. 

Referring to research on clinical samples, the results are similar. For example, Cohen and Kendall [41], examining a group of 90 adolescents in treatment for social phobia and other anxiety disorder, indicated that overt victimization (e.g., relational victimization) predicted the onset of social anxiety, loneliness and depressive symptoms. Regarding the possible psychological moderators of relationship between peer victimization and the onset of social anxiety, interesting results concern the role of personality factors, like neuroticism, and the role of contingent self-worth. Indeed, in study of Calvete et al. [51], adolescents with high level of neuroticism and low level of extroversion would respond to victimization with increased symptoms of depression and social anxiety compared to adolescents with low neuroticism and high level of extraversion. This data is significant for girls but not for boys. Thus, for the girls, high levels of extraversion could be protective factors respect to the onset of social anxiety when they experienced bullying victimization. One possible explanation for this finding is that extraverted girls, relative to extraverted boys, are more likely to have relational orientation styles and seek support in their social networks when they experience environmental stressful events [61]. In addition, high level of contingent self-worth would enhances the effect of victimization on internalizing symptoms like social anxiety [57]. This result is understandable if we define contingent self-worth like the extent to which individuals founded their self-worth and self-esteem on external factors and the perceptions of others [62]. In adolescence, hyper-awareness of others’ perceptions becomes important making adolescents particularly vulnerable to the negative influence of peer victimization on social anxiety.

Overall, the critical analysis of these studies, although conducted with different methodologies (e.g., cross sectional, longitudinal) and types of samples (community sample vs. clinical sample), suggests that being bullied is one of the most stressful experiences for adolescents, especially when it occurs over a prolonged period of time [63]. Victims may experience helplessness and hopelessness. They can be at risk for depression and suicide and may suffer from a lack of an effective strategy to cope with bullying, experiencing sadness and their self-esteem may be invalidated.

The learned helplessness theory explain why victimization of bullying increases the risk of internalizing symptomology in youths [64]. In fact, when people are exposed to persistent negative situations, they start to perceive these events as out of control and they begin to think, feel, and act as if they are helpless. Consequently, there is a refusal to start new behaviors; the subject cannot perceived a right association between his actions and their consequences; he experienced frustration and compared internalizing symptoms, such as depression. Experiencing victimization of bullying may also increase adolescents’ levels of anticipatory anxiety of being bullied again in socially interactional situations, which increases the risk of developing social anxiety.

## 6. Limitations

Some limitations should be considered in our review. Firstly, there is a discrepancy between the included studies on study design, definition of constructs (e.g., peer victimization vs. bullying), and measures for clinical assessment. Based on the definition of constructs, many studies referred indistinctly to bullying and peer victimization, in contradiction with the recent literature [4] that distinguishes the two constructs based on the asymmetric power present in the relationship between bully and victim. This did not allow a quantitative analysis of the results. Secondly, most studies used self-report assessment tools and did not conduct an assessment based on judgment of clinical specialists. This limits the interpretability of the findings. 

## 7. Implications for Practice and Research

The results of this selective review imply that, during the assessment of SAD in children and adolescents, it is important to investigate the presence of peer victimization experiences to facilitate case conceptualization. In addition, children and adolescents with SAD may be supported with a Cognitive Behavioural Therapy (CBT) approach, in order to enhance critical insight and recognition of cognitive biases that determine anticipatory anxiety to support the interactions with peers and prevent the possible avoidance. Still in a cognitive-behavioral perspective, reinforce in children and adolescents with SAD adequate coping strategies could decrease fear of others’ negative judgment and avoidant behavior. 

In addition, the significant social and individual consequences of peer victimization on development of children and adolescents requires prevention strategies. Regarding this, school staff members, especially teachers, have a fundamental role in working to prevent and intervene in school peer victimization. Indeed, rigorous anti-victimization school program are need to increase social skills in classrooms as well as defending behaviors and self-efficacy among scholars [65]. Finally, the strength of this review is not only to investigate the relationship between peer victimization and the onset of social anxiety but also to examine the role of psychological moderators of this relationship. Indeed, to consider the role of neuroticism and higher levels of contingent self-worth in contributing to reactive anxiety symptoms that characterize the victims could be useful in the development of clinical therapeutic programs.

## Figures and Tables

**Table 1:** Results of included studies.

Study	Sample	Study Design	Measure	Results	Limitations
Cohen and Kendall (2014) [41]	*N*: 90 Mean age: 11.06 (± 3.09)	Experimental study	Asher Loneliness Scale ADIS-C/P, CDI, CGAS, ERC, SPPC, MASC, SEQ-S, TQ-R	Overt victimization significantly predicted social anxiety (*p* = 0.003) and loneliness (*p* = 0.001); Relational victimization added significantly to the prediction of social anxiety (*p* = 0.002), depressive symptoms (*p* = 0.001), and loneliness (*p* < 0.001).	Study design: cross-sectional; Measures of victimization are not specific.
Davis (2017) [42]	*N*: 31 Mean age: 14	Experimental study	AWEQ, MASC-2, GBS, MIBI	White accusation was significantly associated with social anxiety symptoms (*p* < 0.01), especially when it is interpreted as a negative evaluation; The accusation was significantly associated with bullying victimization (*p* < 0.05).	Only female participants; Small and specific sample.
Gren-Landell et al. (2011) [39]	*N*: 3211 Mean age: 17.3	Cross-sectional study	SPSQ-C, JVQ	Significant association between SAD and high levels of peer victimization (*p* < 0.001); Females reported significantly more victimization of maltreatment, sexual victimization and victimization from peers and siblings (*p* < 0.001); Males reported a significant difference on the domain of peer/siblings victimization (*p* < 0.05).	Use of self-report instruments; Study design: cross-sectional.
Hamilton et al. (2016) [43]	*N*: 410 Mean age: 12.84	Longitudinal study	CDI, MASC, ALEQ, SEQ-S, CTQ	Peer victimization, interpersonal stressors and emotional maltreatment predicted social anxiety and depressive symptoms.	The findings are not generalizable; Use of self-report instruments; No inclusion of a measures of overt victimization.
Pabian and Vandebosch (2015) [40]	N: 2128 Mean age: 13.02	Longitudinal study	Self-Reported Cyberbullying Behavior, Self-Reported Traditional Bullying Involvement, SAS-A	At baseline social anxiety had a positive correlation with victimization of traditional bullying (*p* < 0.001), victimization of cyberbullying (*p* < 0.001), and perpetration of traditional bullying (*p* < 0.001); These aspects correlated positively with social anxiety 6 months later; Adolescents who scored higher on social anxiety had the same possibility to be victimized 6 months later that adolescents who scored lower	Use of self-report instruments; High drop-out rates; Measures of victimization are not specific.
Quinlan et al. (2018) [44]	*N*: 682	Longitudinal study	OB/VQ, DAWBA, SDQ, CTQ, LEQ	Chronic adolescent peer victimization impact structural brain development and were related to psychopathology symptoms in late adolescence (depression: *p* = 0.001;generalized anxiety: *p* = 0.006); Chronic peer victimization was associated with steeper decreases in left putamen volume (*p* = 0.037) and these changes were also negatively associated with generalized anxiety (*p* = 0.020).	No information about peer victimization or early life stressors before age 14; No analysis of other brain regions linked to chronic peer victimization.
Ranta et al. (2013) [45]	*N*: 3278 Mean age: 15.5	Longitudinal study	SPIN, BDI	Boys’ group there was a bidirectional association between direct peer victimization and social phobia (*p* < 0.05); Girls’ group, the relation victimization predicted the onset of social phobia (*p* < 0.001)	Use of self-report instruments; High drop-out rates.
Rudolph et al. (2016) [46]	*N*: 47	Experimental study	SEQ-S, SMFQ, SAS-A, BIS, Social Achievement Goals Survey, NFA	Victimized girls reported higher levels of depressive symptoms (*p* < 0.001), social anxiety (*p* < 0.01), behavioral inhibition (*p* < 0.001) and avoidance-oriented need for approval (*p* < 0.05); Victimized girls had greater activation of the dorsal anterior cingulate cortex (dACC), the subgenual anterior cingulate cortex (sgACC) and anterior insula Activation to exclusion in the social pain network was associated with internalizing symptoms (*p* < 0.001).	No inclusion of a task measuring neural activation to other types of interpersonal stressors.
Van Oort et al. (2011) [47]	*N*: 2220 Age range: 10–12	Longitudinal study	RCADS, PSI, EMBU-C, YSR, WISC-R, Family History Interview	Victimization experience was correlated to the presence of social phobia in late adolescence (*p* < 0.001); A moderate correlation with social phobia has been found in correlation with being a bully, experienced parents’ mental and physical health problems and the sociometric status (*p* < 0.05).	Not clinical sample; Not exhaustive analysis of risk indicator.
Wu et al. (2016) [48]	*N*: 2872	Cross-sectional study	SASC-R, SPAI-C, CES-DC	Defending behaviours were positively associated with social anxiety and depressive symptoms (*p* = 0.005) in victims and bystanders; Victims and bully-victims also had greater social anxiety (*p* < 0.001) than bullies.	Study design: cross-sectional; No analysis about association of particular form of bullying, defending behaviors and mental health.
Yen et al. (2014a) [49]	*N*: 5252	Cross-sectional study	BMI, C-SBEQ, SPIN, MC-CES-D, K-SADS-E, RSES	The severities of victimization of passive, active bullying and perpetration of passive bullying were positively associated with the severities of social phobia, depression and suicidality (*p* < 0.001); BMI was positively associated with the severities of victimization of passive (*p* < 0.001), active bullying (*p* < 0.01) and perpetration of passive bullying (*p* < 0.05).	Study design: cross-sectional; The data provided by the adolescents themselves; The findings are not generalizable.
Yen et al. (2014b) [50]	*N*: 1604	Cross-sectional study	C-SBEQ, MC-CES-D, MASC-T, SPIN, ADHDS, K-SADS-E, CRAFFT	Victim of passive or active bullying and perpetrator of passive bullying were significantly associated with mental health problems, including social phobia (*p* < 0.001); Perpetrators of only active bullying reported less severe general anxiety and social phobia than perpetrators of only passive bullying (*p* < 0.001); Victims of both passive and active bullying had higher risks of social phobia (*p* < 0.001), depression (*p* < 0.001) and inattention (*p* < 0.001) than victims of only passive or active bullying.	Study design: cross-sectional; The data provided by the adolescents themselves; Different periods of measure about the experiences of bullying and mental health problems.

ADHDS: Attention-Deficit/Hyperactivity Disorder Self-rated Scale, ADIS-C/P: Anxiety Disorders Interview Schedule for Children: Child and Parent Versions, ALEQ: The Adolescent Life Events Questionnaire, AWEQ: The Acting White Experiences Questionnaire, BDI: Beck Depression Inventory, BFQ-C: Big Five Questionnaire-Children, BIS: Behavioral Inhibition Scale, BMI: Body mass index, CES-D: Center for Epidemiological Studies Depression Scale, CES-DC: Center for Epidemiological Studies Depression Scale for Children, CDI: Children’s Depression Inventory, CGAS: Children’s Global Assessment Scale, C-SBEQ: Chinese version of the school bullying experience questionnaire, CRAFFT: Alcohol abuse screening test, CTQ: Childhood Trauma Questionnaire, DAWBA: Developmental and Well-Being Assessment, EMBU-C: Egna Minnen Betraffande Uppfostran for Children, ERC: The Emotion Regulation Checklist, GBS: The Gatehouse Bullying Scale, K-SADS-E: Epidemiological version of the Kiddie Schedule for Affective Disorders and Schizophrenia, LEQ: The self-report Life Events, JVQ: The juvenile victimization questionnaire Questionnaire, MASC: Multidimensional Anxiety Scale for Children, MASC-2: The Multidimensional Anxiety Scale for Children 2nd Edition, MASC-T: Taiwanese version of the Multidimensional Anxiety Scale for Children, MC-CES-D: Mandarin Chinese version of the center for epidemiological studies-depression scale, MIBI: The Multidimensional Inventory of Black Identity, MINI-KID: Mini International Neuropsychiatric Interview for Children and Adolescents, NFA: Need for Approval Questionnaire, OB/VQ: Olweus Bully/Victim Questionnaire, PEQ -VS: Peer Experiences Questionnaire - Victimization of Self scale, PSI: Parental Stress Index, SAS-A: Social Anxiety Scale for Adolescents, SASC-R: Social Anxiety Scale for Children, SCAS-C: Spence Children’s Anxiety Scale: Child Versions, SCAS-P: Spence Children’s Anxiety Scale: Parent Versions, SDQ: Strengths and Difficulties Questionnaire, SEQ-S: Social Experiences Questionnaire-Self-Report, SMFQ: Short Mood and Feelings Questionnaire, SPAI-C: Social Phobia and Anxiety Inventory for Children, SPIN: Social phobia inventory, SPPC: Harter’s Self-perception Profile for Children, SPSQ-C: The social phobia screening questionnaire for children, RCADS: Revised Child Anxiety and Depression Scale, RSES: Rosenberg self-esteem scale, TQ-R: Teasing Questionnaire-Revised, WISC-R: Revised Wechsler Intelligence Scales for Children, YSR: Affective Problems Scale of the Youth Self-Report.

**Table 2:** Results of included studies.

Study	Sample	Study Design	Measure	Results	Limitations
Calvete et al. (2016) [51]	*N*: 1440 Mean age: 13.54	Longitudinal study	BFQ-C CES-D SAS-A	Extraversion girls had lower predictive association between victimization and social anxiety; Adolescents with high levels of extraversion presented a greater reduction in depressive symptoms; Neuroticism predicted both depression and social anxiety, but no significant interactions were evident between neuroticism and bullying victimization; The association between bullying victimization and social anxiety was stronger for boys than for girls (*p* < 0.001); The association between neuroticism and depression was stronger for girls (*p* < 0.001).	Use of self-report instruments; No differentiation between direct and indirect; Use few variables.
Early et al. (2017) [56]	*N*: 154 clinical sample *N*: 116 community sample Mean age: 13.07	Cross-sectional, quantitative survey	MINI-KID SCAS-C SCAS-P Peer acceptance 3 items PEQ -VS	Significant group differences in participant self-rated social acceptance (*p* < 0.001), parent-rated social acceptance (*p* < 0.001), and relational victimization (*p* < 0.05); Social anxiety group presented lowest social acceptance compared to other anxiety group and the community sample; Girls in the community sample showed more overt victimization than girls with SAD and other anxiety diagnoses; The results showed significant interaction between group status and self-reported social anxiety (*p* < 0.05) and nonsocial anxiety (*p* < 0.05) in predicting relational victimization in the community sample.	Use of rating scale without behavior observation.
Ghoul et al. (2013) [57]	*N*: 716 Mean age: 15.95	Experimental study	RCDAS SWCQ Exposure to School Aggression Scale	Results showed that victimization and contingent self-worth were positively correlated with social phobia (*p* < 0.001); Higher levels of contingent self-worth amplify the effect of victimization on internalizing problems; For social phobia, this effect appeared to be salient only for boys.	Use of self-report instruments; No longitudinal data.
Silberg et al. (2016) [58]	*N*: 145 pairs Age range: 8–17	Longitudinal study	CAPA SCID	Bullied MZ twins compared to their non-bullied co-twin have the higher rate of social anxiety (27%), separation anxiety (15%), Attention Deficit and Hyperactivity Disorder (5%), and young adult suicidality (12%); Significant individual-specific environmental correlations between bullying victimization and social anxiety, separation anxiety and young adult suicidality; Highly significant association between early bullying victimization and later social anxiety (*p* < 0.001) and bullying victimization and later young adult suicidality (*p* < 0.05); Genetic factors were also influential in bullying victimization and social anxiety.	Need to replicate the data.
Spence et al. (2017) [59]	N: 6310 Age range: 4–17		DISC-IV Social envirometal dataImpact of functioning	Social anxiety disorder, separation anxiety disorder and generalized anxiety disorder were all associated with having a parent with a mental health problem and repeated bullying over the previous 12 months (*p* < 0.001).	Not analysis of lifetime presence of disorders; Use parental report.

BFQ-C: Big Five Questionnaire-Children; CAPA: Child and Adolescent Psychiatric Assessment; CES-D: Center for Epidemiological Studies Depression Scale; DISC-IV: Diagnostic Interview Schedule for Children–Version IV; MINI-KID: Mini International Neuropsychiatric Interview for Children and Adolescents; SAS-A: Social Anxiety Scale for Adolescents; SCAS-C: Spence Children’s Anxiety Scale: Child Versions; SCAS-P: Spence Children’s Anxiety Scale: Parent Versions; SCID: Structured Clinical Interview for DSM; PEQ -VS: Peer Experiences Questionnaire - Victimization of Self scale; RCADS: Revised Child Anxiety and Depression Scale; SWCQ: Self-Worth Contingency Questionnaire.

## Supplementary Materials

The following are available online at https://www.mdpi.com/2076-3425/9/6/132/s1, Table S1: Search Strategy.
