# Supplementary material for: Peer Victimization and Onset of Social Anxiety Disorder in Children and Adolescents
Source: Brain Sci. 2019 Jun 6;9(6):132. doi: 10.3390/brainsci9060132 (PMC6627045; doi:10.3390/brainsci9060132)
Supplement: Supplementary file 1 [file brainsci-09-00132-s001.pdf]

Supplementary Information

Peer Victimization and Onset of Social Anxiety Disorder in Children and Adolescents

Maria Pontillo *, Maria Cristina Tata, Roberto Averna, Francesco Demaria, Prisca Gargiullo, Silvia Guerrera, Maria Laura Pucciarini, Ornella Santonastaso and Stefano Vicari

Child and Adolescence Neuropsychiatry Unit, Department of Neuroscience, Children Hospital Bambino Gesù, Piazza Sant'Onofrio 4, 00165 Rome, Italy; mariacristina.tata@opbg.net (M.C.T.); roberto.averna@opbg.net (R.A.); francesco.demaria@opbg.net (F.D.); prisca.gargiullo@opbg.net (P.G.); silvia.guerrera@opbg.net (S.G.); laura.pucciarini@gmail.com (M.L.P.); ornella.santonastaso@libero.it (O.S.); stefano.vicari@opbg.net (S.V.)

* Correspondence: maria.pontillo@opbg.net; Tel.: +39-0668-592030

Table S1. Search Strategy.

Search	Query	Items found
Recent queries in Pubmed on January 18, 2019		
1	Bullying	5660
2	Peer victimization	1787
3	((bullying OR peer victimization)) AND social phobia	28
4	((bullying OR peer victimization)) AND (social phobia OR social anxiety disorder)	109
5	(((bullying OR peer victimization)) AND (social phobia OR social anxiety disorder)) AND (children AND adolescents)	58
6	(((((bullying OR peer victimization)) AND (social phobia OR social anxiety disorder)) AND (children AND adolescents))) AND ("2011"[Date - Publication] : "2018"[Date - Publication])	50
Recent queries in Cochrane Library database on January 18, 2019		
1	(((((bullying OR peer victimization)) AND (social phobia OR social anxiety disorder)) AND (children AND adolescents))) AND ("2011"[Date - Publication] : "2018"[Date - Publication])	8
Recent queries in Cinhal Complete on January 18, 2019		
1	(((((bullying OR peer victimization)) AND (social phobia OR social anxiety disorder)) AND (children AND adolescents))) AND ("2011"[Date - Publication] : "2018"[Date - Publication])	10
Recent queries in Scopus on January 18, 2019		
1	(((((bullying OR peer victimization)) AND (social phobia OR social anxiety disorder)) AND (children AND adolescents))) AND ("2011"[Date - Publication] : "2018"[Date - Publication])	61
